# Postoperative word-finding difficulties in children with posterior fossa tumours: a crosslinguistic European cohort study

**DOI:** 10.1007/s00381-025-06787-4

**Published:** 2025-03-12

**Authors:** K. Persson, J. Grønbæk, I. Tiberg, Å. Fyrberg, C. Castor, B. Andreozzi, R. Frič, P. Hauser, R. Kiudeliene, C. Mallucci, R. Mathiasen, P. Nyman, B. Pizer, A. Sehested, D. Boeg Thomsen, The CMS study group

**Affiliations:** 1 Department of Health Sciences, Lund University, Lund, Sweden; 2 Department of Paediatric and Adolescent Medicine, Copenhagen University Hospital Rigshospitalet, Copenhagen, Denmark; 3 Department of Speech and Language Pathology, University of Gothenburg, Gothenburg, Sweden; 4 Department of Oncology/Hematology, Cell Therapy Gene Therapies and Hemopoietic Transplant, Scientific Institute for Research, Hospitalization and Healthcare Bambino Gesù Children’s Hospital, Rome, Italy; 5 Department of Neurosurgery, Oslo University Hospital Rikshospitalet, Oslo, Norway; 6 Department of Pediatrics, Semmelweis University, Budapest, Hungary; 7 Faculty of Health Care, University of Miskolc, Miskolc-Egyetemváros, Hungary; 8 Department of Pediatrics, Lithuanian University of Health Sciences, Kaunas, Lithuania; 9 Department of Neurosurgery, Alder Hey Children’s NHS trust, Liverpool, UK; 10 Department of Clinical Medicine, Copenhagen University Hospital Rigshospitalet, Copenhagen, Denmark; 11 Crown Princess Victoria Children’s Hospital and Department of Biomedical and Clinical Sciences, Linköping University, Linköping, Sweden; 12 University of Liverpool, Liverpool, UK; 13 Department of Nordic Studies and Linguistics, University of Copenhagen, Copenhagen, Denmark; 14 Department of Neurosurgery, Copenhagen University Hospital Rigshospitalet, Copenhagen, Denmark

**Keywords:** Cerebellar mutism syndrome, Child language, Language impairment, Postoperative speech impairment (POSI), Posterior fossa tumour, Word-finding difficulties

## Abstract

**Purpose:**

Posterior fossa tumour (PFT) surgery carries a risk of mutism or severely reduced speech. As for higher-cognitive language functions, word-finding difficulties have been reported, but no study has compared pre- and postoperative word-finding speeds to identify impairment caused by surgery. The current study investigated changes in word-finding ability associated with PFT surgery and examined factors affecting postoperative ability.

**Method:**

We included 184 children aged 5:0–17:9 years undergoing PFT surgery and assessed word-finding ability before and after surgery using a speeded picture-naming test. We compared postoperative word-finding performance with both preoperative performance and age-specific norms and examined factors affecting word-finding ability.

**Results:**

We found no significant difference between pre- and postoperative performance, reflecting that some children exhibited better word-finding ability after surgery, others poorer. After surgery, 95% of the children performed two standard deviations above (slower than) age-specific norms. Tumour location in the fourth ventricle negatively affected postoperative word-finding ability (*B* = −4.09, *p* < 0.05).

**Conclusion:**

For some children, PFT surgery leads to postoperative word-finding difficulties, emphasizing the importance of postoperative language assessments and interventions. Fourth-ventricle tumour location emerged as a risk factor for poorer postoperative word-finding ability, likely reflecting surgical damage to the dentato-thalamo-cortical pathway (DTCP).

## Introduction

Posterior fossa tumours comprise about half of all central nervous system tumours in children [1]. Surgical resection is essential, aiming to cure the patient and to alleviate the burden of acute symptoms, but carries a risk of complications, notably the development of cerebellar mutism syndrome (CMS) in approximately a quarter of affected children [2]. CMS is characterized by mutism or severely reduced speech, denoted as postoperative speech impairment (POSI) [3], emotional lability and other neurological deficits [4]. Symptoms typically have a delayed onset, emerging on average around 2 days post-surgery but can appear anywhere from the day of surgery up to 15 days later [5]. The condition can last weeks to months and can lead to persistent speech, language and/or communication impairments [4, 5]. POSI can evolve into a motor speech disorder, often described as dysarthria, with slow speech rate, monotonous tone and ataxic speech [6, 7]. Language impairments following POSI are still poorly understood with current studies suggesting that these impairments may involve both comprehension and production, affecting both morphosyntax and lexical semantics [8–10]. Even children who do not suffer from POSI may exhibit postoperative language impairments [10, 11]. The severity can vary, suggesting a continuum of postoperative language impairments [12, 13]. This study focuses on a specific lexical ability: word-finding ability. Word-finding ability refers to the capacity to quickly and accurately retrieve the appropriate word from the mental lexicon [14]. Word finding involves several processes: the semantic process (retrieving words as units of meaning), the phonological process (retrieving words as structures of speech sounds) and speech processes for planning and executing articulation [15, 16]. It further depends on general processing speed [17]. The cerebellum is suggested to be involved in each of these processes [18–20], and impairments in any or a combination of these may contribute to word-finding difficulties in children with posterior fossa tumour (PFT). PFTs have been shown to be associated with word-finding difficulties both pre- and postoperatively [8, 21, 22], with preoperative difficulties linked to the tumour itself and postoperative impairments primarily attributed to the effects of surgery. Di Rocco et al. (2011) reported that 12% of the children undergoing PFT surgery in their sample experienced postoperative word-finding difficulties [8]. However, the test they used for assessing word-finding abilities (Boston Naming Test) [23] only measured word-finding accuracy, not speed, which, as previously mentioned, is a crucial aspect of the word-finding process [15–17], and the actual prevalence of postoperative word-finding difficulties may thus be much higher. As for lexical-semantic difficulties in general, the review by Svaldi et al. highlights that the quality of the current available evidence is low, and that there is no clear understanding of the nature or duration of impairments post-surgery. They conclude that postoperative difficulties are common, but the prevalence and severity vary across studies [24]. Children with tumours in the right cerebellar hemisphere or brainstem have been shown to be at higher risk of having preoperative word-finding difficulties [21, 22]. The finding of an effect of right cerebellar hemisphere location supports theories of functional cerebellar topography and cerebellar language lateralization, which propose the involvement of this hemisphere in linguistic processing, including word-finding tasks [25–28]. To our knowledge, only one study has investigated risk factors for postoperative word-finding difficulties [22]. As part of a larger study of neurological, neuropsychological and behavioral impairment in children undergoing PFT surgery, di Rocco et al. found that postoperative word-finding difficulties were associated with invasion of the brainstem and the right dentate nucleus. No association was found for tumour type, preoperative hydrocephalus or location (operationalized coarsely as midline vs. hemispheric) [22]. While the study carefully investigated both pre- and postoperative word-finding abilities, preoperative performance was not used as a baseline for analyzing postoperative change, and it is thus uncertain to which degree the factors associated with postoperative word-finding difficulties were related to surgery or had already caused poor word-finding abilities before surgery. Further, as this study too depended on the use of the Boston Naming Test for evaluating word-finding ability, factors potentially affecting word-finding speed could not be investigated. For language impairment more broadly, the review by Svaldi et al. found tumour location in the brainstem or in the fourth ventricle to be associated with a high risk of postoperative linguistic impairment, but as they stressed, this observation depended on a small sample size [24]. For POSI, tumours involving the right dentate nucleus, brainstem compression or infiltration, fourth ventricle tumours, diagnosis of medulloblastoma and younger age have been identified as significant risk factors [3, 29]. For medulloblastomas, molecular profiling has advanced classification beyond histology, identifying four subgroups: wingless (WNT), sonic hedgehog (SHH), group 3 and group 4. Group 3 and group 4 tumours, often midline and larger, carry a higher risk of CMS, while SHH tumours have the lowest risk [30]. For surgical approach, the evidence is mixed. The telovelar surgical approach has been suggested to carry a lower risk of CMS compared to the transvermian approach [31–35], but the largest studies of posterior fossa tumours in children did not identify an association between surgical approach and the risk of POSI [3, 36]. Furthermore, larger tumour volume has been proposed as a risk factor, with more extensive lesions potentially requiring greater surgical manipulation and increasing the risk of CMS [36, 37]. Preoperative hydrocephalus is less well investigated [38] and may not directly cause POSI, but could exacerbate the severity of the condition [22, 39]. Word-finding difficulties might occur on a continuum with children suffering from POSI at one end of the continuum and children with mild word-finding difficulties at the other, and risk factors for more severe degrees of impairment need exploration. Understanding the continuum of difficulties affecting children postoperatively has potential to inform the development of targeted assessments and rehabilitation interventions.

In the current study, we aimed to:

1. Investigate if children with PFT exhibit postoperative word-finding difficulties. We hypothesized that children with PFT would exhibit significant impairments in word-finding abilities compared to age-specific norms.
2. Investigate changes in word-finding performance from pre- to postoperative testing in children with PFTs. We hypothesized that children would experience an impairment in word-finding ability postoperatively, with a worse degree associated with dysarthria and POSI.
3. Investigate factors affecting postoperative word-finding ability. We hypothesized that right-hemisphere, brainstem and fourth ventricle involvement would be associated with an elevated risk of postoperative word-finding difficulties compared to other tumour locations. For the remaining factors (other tumour location, tumour histology, preoperative hydrocephalus, sex and age), the analysis was explorative.

## Methods

### Study design and settings

The present study is an observational cohort study conducted as part of the Nordic-European CMS study, which has been described elsewhere [40]. Children who underwent tumour surgery in the posterior fossa in Austria, Czech Republic, Denmark, Finland, Germany, Hungary, Italy, Lithuania, Norway, Sweden, The Netherlands and UK between 2014 and 2024 were included. Regional and national ethics committees approved the study.

### Participants

**Fig. 1:**
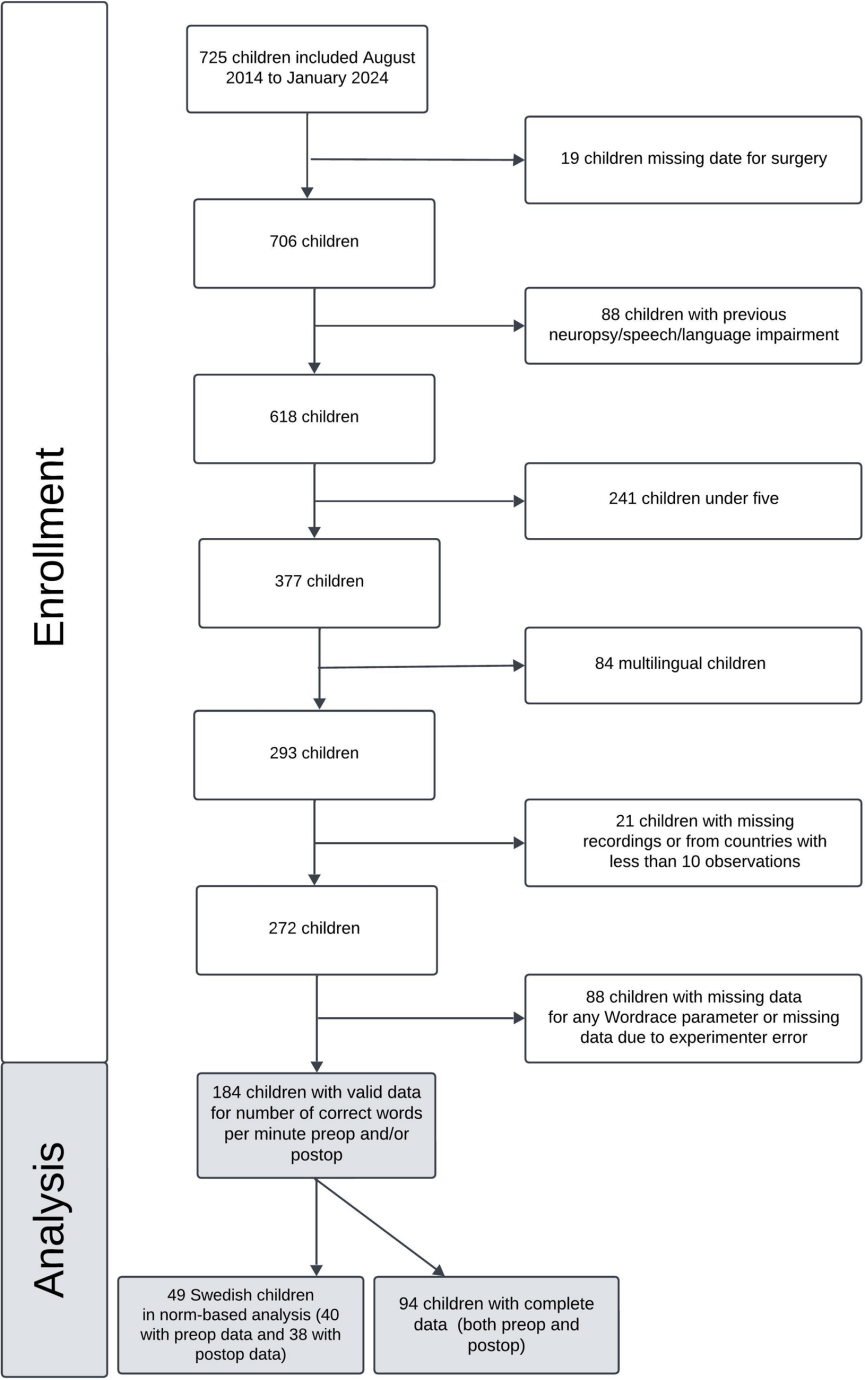
The inclusion process and the children included in each analysis.

Between August 2014 and January 2024, 725 children aged below 18 years were included in the Nordic-European CMS study. To minimize the influence of any confounding factors, we excluded those with additional diagnoses such as neuropsychological disorders (e.g. autism, ADHD), previously reported speech and language disorders, previous tumour surgery, children younger than 5 years (due to lack of norm data), multilingual children and those from countries with fewer than 10 observations. Invalid data due to experimenter error were also discarded. The final sample included 184 children from Sweden, Denmark, Norway, Italy, Lithuania, UK and Hungary with valid word-finding data pre- and/or postoperatively. Of these, 49 Swedish children were included in a subanalysis using Swedish age-specific word-finding norms. Figure 1 presents the inclusion process, and the children included in each analysis.

### Materials

The 25-item picture-naming test Wordrace was used to assess word-finding ability. The instrument was developed for the Nordic-European CMS study to measure word-finding ability, measured as *total number of correct words* and *total test time* [40, 41]. Details on Wordrace can be found in previously published online supplemental materials [21]. Currently, normative data for Wordrace is available for the Swedish language in a master’s thesis involving 299 typically developing Swedish children aged 5–15 years. Results showed a negative correlation between total test time and age (i.e. older children name the items faster). Most children in the study completed the test without errors [42]. Given that language- and age-specific norms are only available for Swedish, we only include data from our Swedish participants in the subanalysis evaluating postoperative word-finding difficulties compared to norms.

### Data collection

Wordrace was administered by a physician, nurse or speech and language pathologist at the pediatric oncology centres. Preoperative tests were conducted close to the surgery date and postoperative tests within 1–4 weeks after surgery. The 25 pictures were presented one at a time, either on a screen or on paper, and children were instructed to name them quickly. The test leader immediately switched to the next picture once a picture was named. If a child failed to name a picture, no additional cues were provided, and the next picture was displayed after 5 s. The test procedure was audio-recorded and stored in the Nordic-European CMS database. Demographic data, including birthdate, sex, presence of dysarthria, tumour location, histology, hydrocephalus and presence and duration of mutism and/or reduced speech, were collected by physicians and stored in the database. Dysarthria was reported on a four-point scale from normal to absent/unintelligible speech as judged by the physician, with separate reports for preoperative and postoperative dysarthria. Tumour location, reported by the surgeon after surgery, could include more than one site in the posterior fossa: left cerebellar hemisphere (LCH), right cerebellar hemisphere (RCH), cerebellar vermis (VR), fourth ventricle (FV) and/or brainstem involvement (BS). Tumour histology was categorized as pilocytic astrocytoma, medulloblastoma, ependymoma, atypical teratoid rhabdoid tumour or other. Data regarding subgroup of medulloblastoma was collected when available as part of standard of care. Hydrocephalus was reported as absent or present, with separate reports for preoperative and postoperative hydrocephalus. Mutism was defined as “absence of speech with no production of words or short sentences, but crying or whining can still be produced in some children”. Reduced speech was defined as “severely reduced speech production limited to single words or short sentences, which can only be elicited after vigorous stimulation”. The number of days of mutism or reduced speech was also reported.

### Coding

Wordrace was scored by speech analysts in each country, with the exception of UK, which was scored centrally. Two parameters were scored separately: total correct words and total test time. This scoring approach entails the risk that children who self-correct may achieve a poorer outcome in total test time despite being linguistically superior to those producing incorrect responses. To avoid this error, the current study integrated the two measures and used the number of correct words per minute as the main outcome. Words per minute was calculated as: (60 × number of correct words) / total test time in seconds. For example, if a child completes the test in 77 s and correctly names 22 words, their score would be approximately 17.14 words per minute. The original measures, total correct words and total test time, were used as outcomes in the analysis utilizing Swedish age-specific norms, as no norms for the integrated measure were available. In analyzing factors affecting postoperative word-finding ability, we investigated the influence of each of the five possible tumour locations separately, treating each location as a binary variable (location involved: yes/no). Tumour histology was classified into three categories: pilocytic astrocytoma, medulloblastoma and other. Molecular subgrouping data for medulloblastomas was not included due to substantial missing data, which rendered further analyses infeasible. Dysarthria was classified as absent or present. Age was calculated by determining the time between the child’s birthdate and date of surgery.

### Statistical analysis

We compared the total number of correct words and the total test time among the Swedish children in the cohort with age-specific Swedish norms. We identified postoperative word-finding difficulties by using cut-off levels of 1 and 2 standard deviations below the Swedish norm. For the second analysis we used a paired t-test to determine a possible difference between the pre- and postoperative correct words per minute measure in children who underwent both pre- and post-op assessments. We used descriptive statistics to describe the relationship between the changes and POSI on the one hand and dysarthria on the other. To analyze factors affecting postoperative word-finding ability, we used linear mixed-effect models. These models incorporate words per minute as outcome, and all factors were examined in interaction with timepoint (pre vs. post) to detect the effect of surgery. Main effects apply to the entire dataset, both pre- and postoperative, and do not indicate the effect of surgery. All models included participants and language as random effects. Observations were excluded if they had missing values for any variables in the model, except for words per minute. The models include cases with only preoperative, only postoperative or both measurements. The models were built in steps, exploring the effects of tumour location, tumour histology, hydrocephalus, sex, age, dysarthria and test format. For the model with multiple independent variables, a backward selection approach was used, starting with the maximal model comprising all predictors. Models were compared using the Akaike Information Criterion (AIC), where lower values indicate a better fit [43]. Statistical analyses were conducted in RStudio (Copyright (C) 2022 by Posit Software, PBC, Version 2023.12.1+ 402). To account for different effects across age, we used a spline technique where we incorporated two knots at distinct age intervals at 7 and 12 years. The knots were chosen based on knowledge of lexical development, with substantial increase in vocabulary exposure in school and reorganization of children’s vocabulary during the early school years and at puberty onset [44, 45].

## Results

Most children were speakers of a Scandinavian language (54%) or English (21.3%). Out of the 184 children, 37 (20.1% ) were reported to have had POSI. Among these, 25 had experienced reduced speech which persisted for 2 to 21 days (median 3 days), and 12 had suffered from mutism which lasted from 1 to 7 days (median 1.5 days). Of the children with POSI, 16 had postoperative dysarthria, equally divided between those with mutism (8) and those with reduced speech (8). Demographic data for all children, for the subset with complete data and for the Swedish subset are presented in Table 1.

**Table 1:** Demographic data for all children, all children with complete data and for all Swedish children

	**All children** ***n =* 184**	**All children with complete data** ***n =* 94**	**All Swedish children** ***n =* 49**
Age (median, Q1, Q3)	9.8, 7.4, 12.9	10.9, 8.0, 13.3	9.8, 7.5, 11.5
Sex, *n* (%)			
Girls	87 (47.3)	47 (50)	23 (46.9)
Boys	97 (52.7)	47 (50)	25 (53.1)
Language, *n* (%)			
Swedish	49 (26.6)	29 (30.6)	–
English	47 (25.5)	20 (21.3)	–
Danish	29 (15.8)	9 (9.6)	–
Norwegian	23 (12.5)	13 (13.8)	–
Hungarian	13 (7.1)	9 (9.6)	–
Lithuanian	12 (6.5)	8 (8.5)	–
Italian	11 (6.0)	6 (6.4)	–
POSI, *n* (%)			
Mute	12 (6.9)	4 (4.8)	4 (9.8)
Reduced speech	25 (14.4)	8 (9.5)	9 (22.0)
Habitual speech	136 (78.7)	72 (85.7)	28 (68.2)
Unknown	11	10	8
Tumour location, *n* (%)			
LCH (yes)	53 (30.1)	28 (31.1)	17 (36.1)
RCH (yes)	64 (36.4)	36 (40.0)	17 (36.1)
VR (yes)	73 (41.5)	31 (34.4)	22 (46.9)
FV (yes)	65 (36.9)	31 (34.4)	20 (42.6)
BS (yes)	33 (18.8)	17 (18.9)	9 (19.1)
Unknown	8	4	2
Tumour histology, *n* (%)			
Pilocytic astrocytoma	85 (51.8)	41 (48.2)	20 (48.8)
Medulloblastoma	51 (31.1)	27 (31.7)	14 (34.1)
Other	28 (17.1)	17 (20.0)	7 (17.1)
Unknown	20	9	8
Dysarthria, pre- postop, *n* (%)			
Present	7 (4.7), 23 (15.4)	4 (4.7), 12 (15.2)	0 (0), 6 (13.6)
Absent	144 (95.4), 126 (84.6)	82 (95.3), 67 (84.8)	44 (100), 38 (86.4)
Unknown	33, 35	8, 15	5, 5
Hydrocephalus, pre- postop, *n* (%)			
Present	121 (67.2),13 (8.0)	63 (69.2), 3 (3.6)	32(65.3) 2 (5.1)
Absent	59 (32.8), 149 (91.9)	28 (30.8), 80 (96.4)	17(34.7) 37 (94.9)
Unknown	4, 22	3, 11	10, 10

*POSI* postoperative speech impairment, *LCH* left cerebellar hemisphere, *RCH* right cerebellar hemisphere, *BS* brainstem, *VR* vermis, *FV* fourth ventricle, *Other* ependymoma, AT/RT and other tumours

### Postoperative word-finding difficulties

**Fig. 2:**
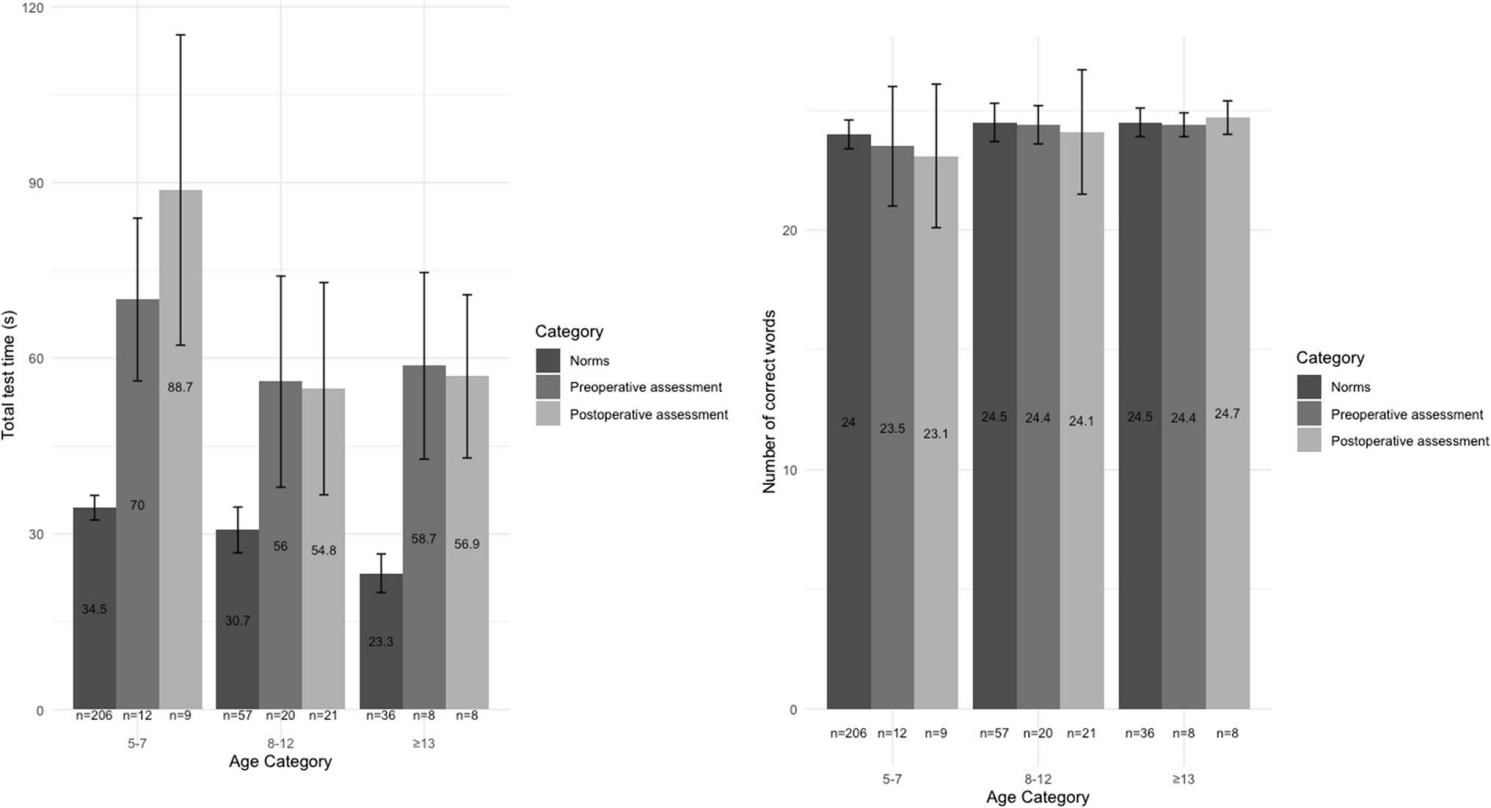
Total test time (left panel) and total correct words (right panel) pre- and postoperatively in Swedish children undergoing tumour surgery in posterior fossa compared with Swedish norms (means and SDs). Total test time is in seconds and total correct words in number of correct responses.

**Fig. 3:**
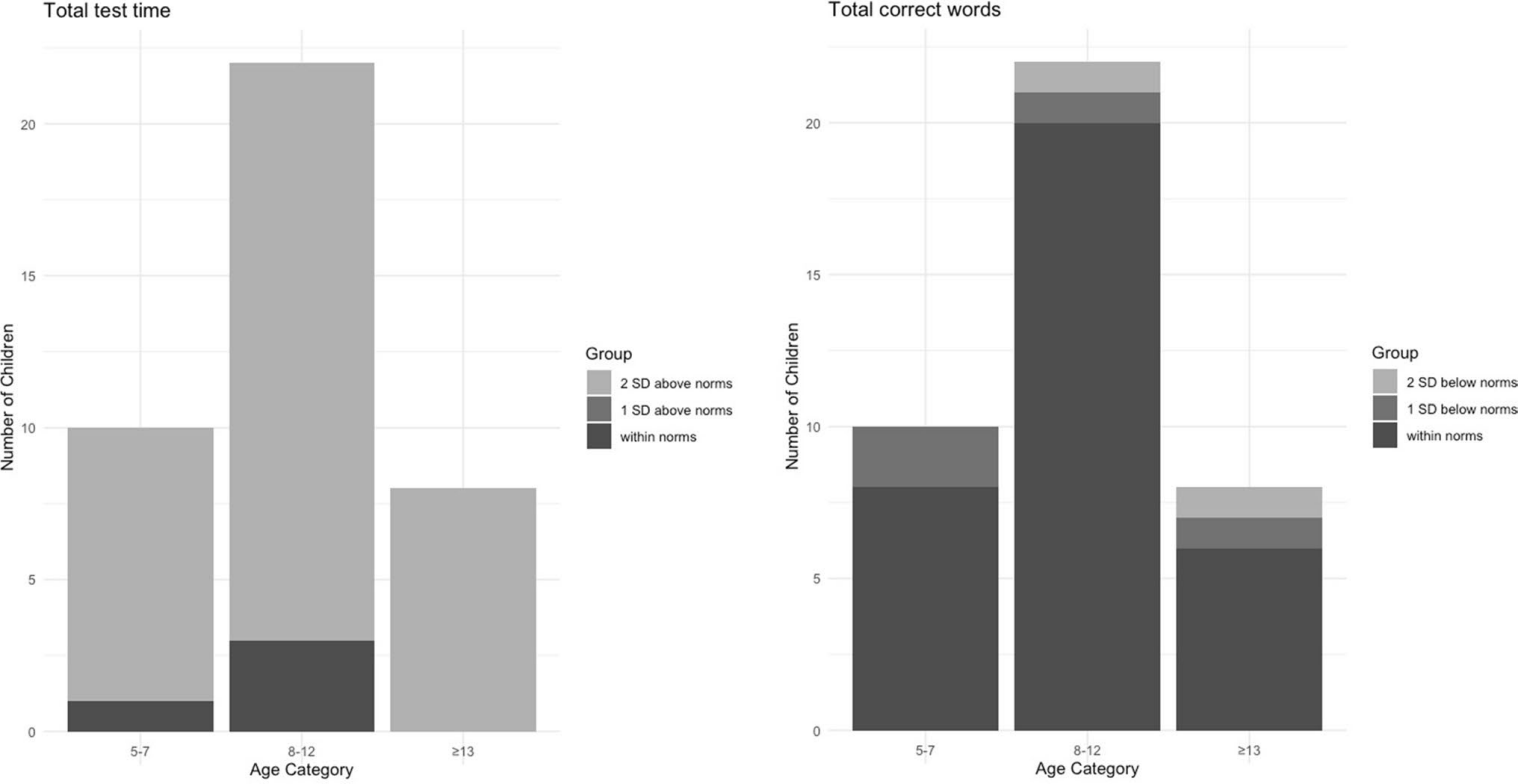
Distribution of postoperative total test time (left panel) and total correct words (right panel) across age categories, differentiated by SDs from age-specific norms.

To identify word-finding difficulties, we compared patients’ performance with norms from typical development, and as language- and age-specific norms for Wordrace are only available for Swedish, this norm-based analysis was conducted on the subset of Swedish children (*n* = 49). Of these, 40 had a preoperative assessment and 38 had a postoperative assessment. We compared both their preoperative and their postoperative performance with norms from typically developing Swedish children (5–9 years: *n* = 206, 10–12 years: *n* = 57, 13–15 years: *n* = 38). As illustrated in Figure 2, children undergoing surgery for PFT exhibited a total test time approximately twice as long as the established norms, both before and after surgery. Postoperatively, 95% of the children (36 out of 38) had a total test time two standard deviations (SDs) or more above the norms (i.e. the children were substantially slower). Of these, one child had experienced a period of mutism, six had experienced a phase of reduced speech and three had postoperative dysarthria (two had unknown motor speech status). When considering the total number of correct words, 16% (6 out of 38) performed one SD below the norms, whereof 5.3% (2 out of 38) exhibited serious impairment, obtaining scores two SDs below the norms. Of these children with serious impairment, none had experienced mutism or reduced speech and one had postoperative dysarthria. Thus, while no marked impairment in accuracy was observed at the group level, impairment was observed in individual children. The distribution of postoperative total test time and total correct words across age categories, differentiated by SDs from norms, are shown in Figure 3. This result indicates a high prevalence of impairments in word-finding speed both before and after surgery in all age groups.

**Fig. 4:**
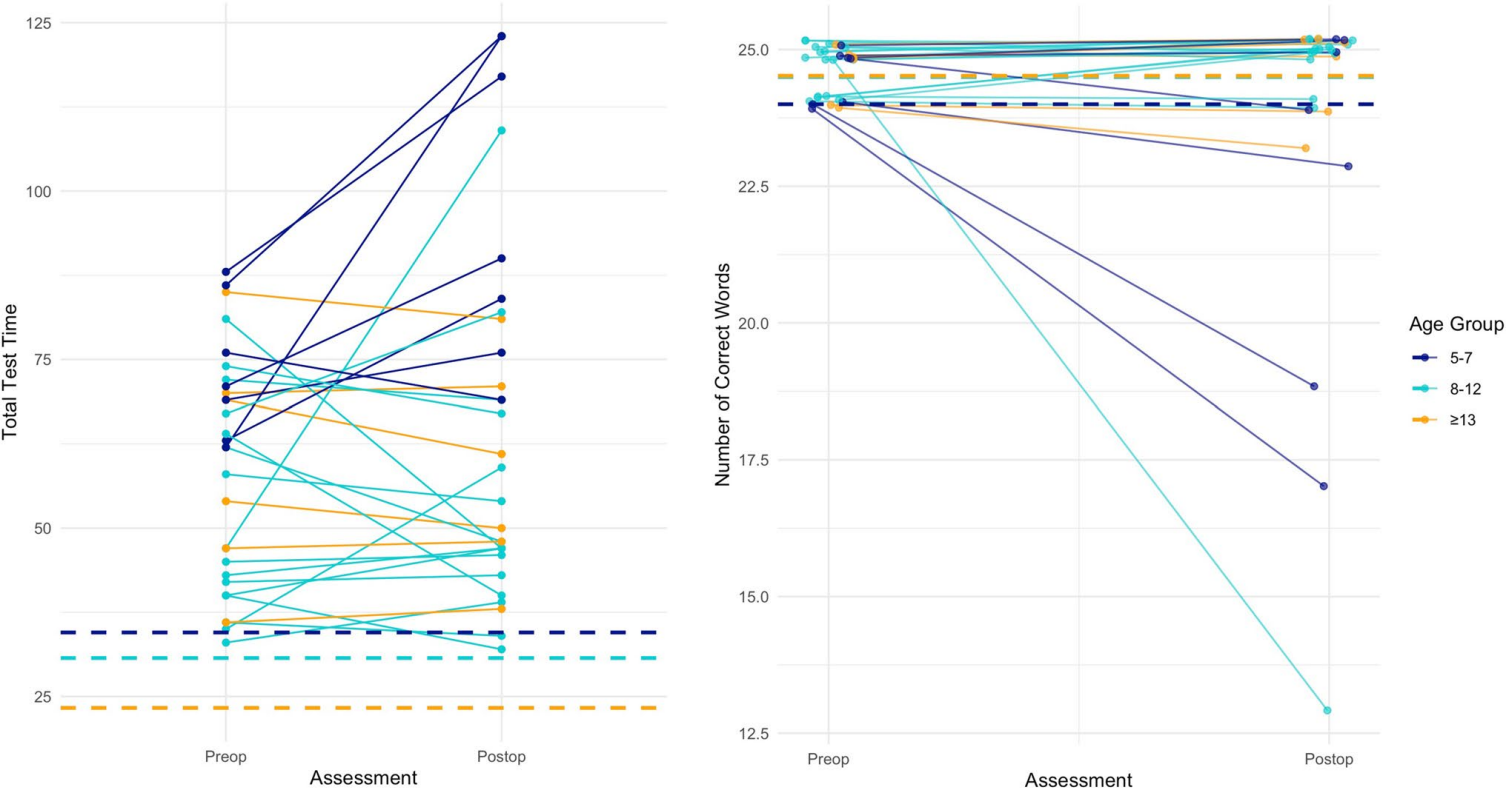
Individual changes in total test time (left panel) and number of correct words (right panel) from pre- to postoperative assessment, compared to age-specific norms. Dashed lines indicate age-specific means, with colours representing the different age groups

As we see severe word-finding impairment also before surgery, it is interesting to inspect whether it is the same children who exhibit difficulties before and after surgery, which could indicate stable impairment unaffected by surgery, or whether we see changes in individual children’s abilities, which would suggest effects of surgery. Figure 4 illustrates individual changes in total test time (speed, left panel) and number of correct words (accuracy, right panel) pre- and postoperatively. While the group means for both measures remain consistently slower (for speed) or lower (for accuracy) compared to the norms, substantial variability is evident among individual children, and it is clear that for some children, performance is much poorer after surgery, while for others, it is much better. Thus, having difficulties before surgery does not necessarily entail more severe difficulties after surgery, and the variability in outcomes suggests that surgery has a negative effect on some children’s word-finding abilities and a positive effect on others’.

### Changes in word-finding ability

**Fig. 5:**
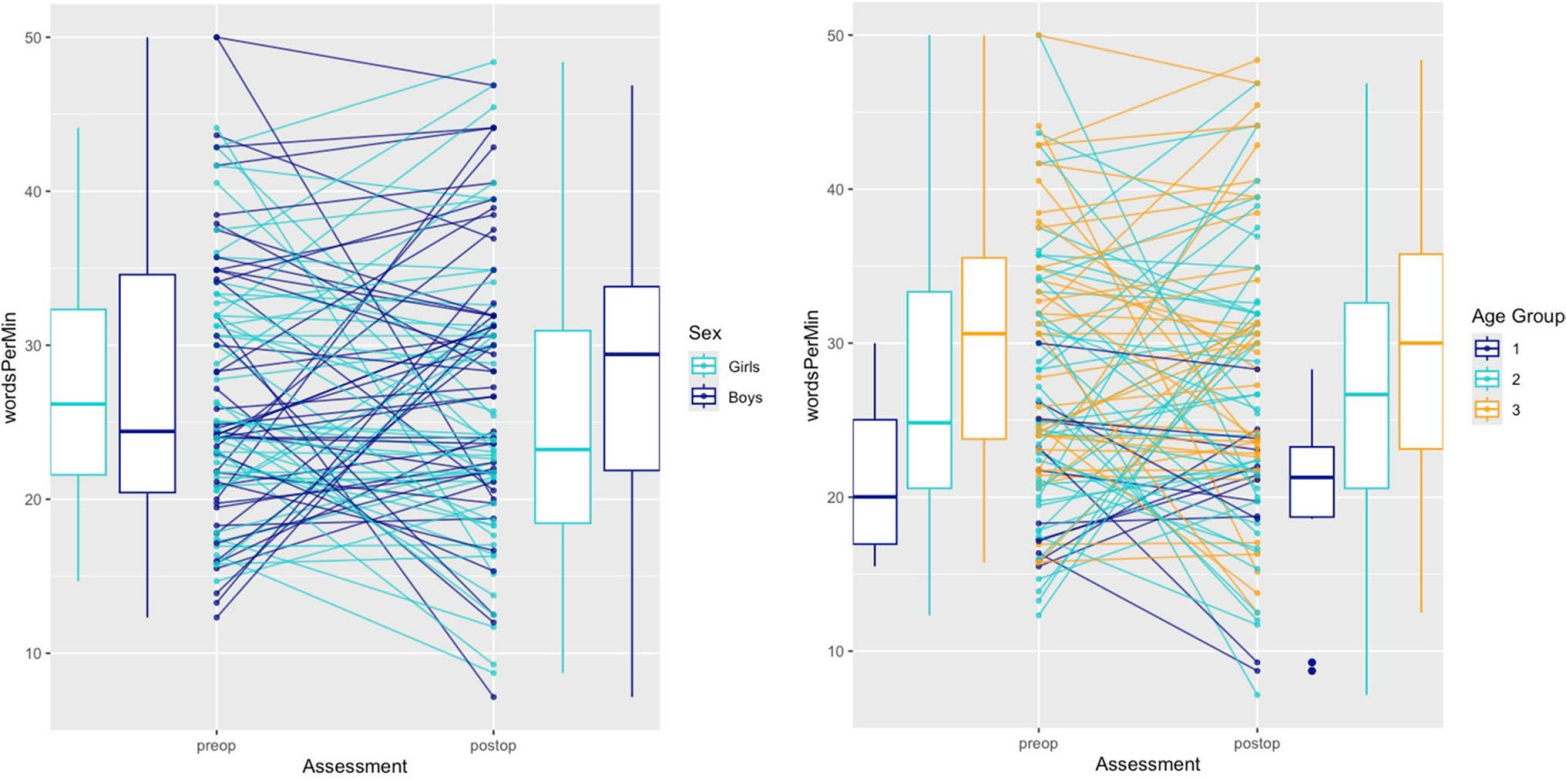
Changes from pre- to postoperative correct words per minute stratified by sex and age groups. Each line represents a child (*n* = 94). The left panel shows changes stratified by sex (boys: dark blue, girls: turquoise) and the right panel by age groups (1, 5–7 years; 2, 8–12 years; 3, ≥13 years). Boxplots summarize the distributions at each assessment point.

Changes in word-finding ability were analysed without relying on norms, allowing inclusion of the full dataset, encompassing all 94 children with complete preoperative and postoperative data. No statistically significant difference was observed (95% CI −1.82 to 2.30,* p* = 0.8191) when comparing pre- and postoperative word-finding ability in terms of correct words per minute. About half of the children (*n* = 45) showed improved word-finding abilities postoperatively, and the other half (*n *= 46) showed a decline, as illustrated in Figure 5. A few (*n* = 3) had unchanged abilities (same number of correct words per minute before and after surgery).

Of the 94 children, 12 had had POSI. Among these, eight (four children with reduced speech and four children with previous mutism) showed a decrease in correct words per minute (mean −11.9, SD 4.8, range −18.1 to −5.7). There was no significant association between the presence of POSI and postoperative decline (Fisher’s exact *p* > 0.05). However, the four children with a period of mutism all showed a postoperative decline (two of them with dysarthria) which was near significant (Fisher’s exact *p* = 0.053). Of the 94 children, 12 had postoperative dysarthria (five with POSI). Among these, nine showed a decrease in correct words per minute (mean −7.6, SD 7.5, range −30.0 to −0.5). There was no significant association between the presence of dysarthria and postoperative decline (*χ*^2^ = 2.64, *p* > 0.05).

Given the extensive variability seen in postoperative outcomes, it is interesting to explore the potential factors underlying this variability, and to do so, the next section turns to regression analysis.

### Factors affecting postoperative word-finding ability

Step 1: Null model

Initially, a null model was created including the dependent variable (correct words per minute; *M* = 27.21, max = 50, SD = 8.92) and the timepoint for assessment.

Step 2: Location of tumour model

To explore the effect of tumour location, the null model was compared with a model including tumour locations (LCH, RCH, BS, VR and FV) as fixed interaction effects with timepoint (pre vs. post). Significant negative impacts on postoperative performance were found for LCH: *B =* −5.22, SE = 1.95, 95% CI −9.06 to −1.38, *p <* 0.01 and FV: *B =* −5.17, SE = 1.89, 95% CI −8.90 to −1.44, *p <* 0.01. The AIC decreased from 1993.65 to 1441.49, indicating improved fit.

Step 3: Histology model

Adding tumour type (medulloblastoma, pilocytic astrocytoma, other) as an interaction effect with timepoint showed no significant impact. The negative impact of tumour location remained significant: LCH: *B* = −5.47, SE = 2.11, 95% CI −9.62, −1.31, *p* < 0.05 and FV: *B* = −4.94, SE = 2.33, 95% CI −9.53, −0.35, *p* < 0.05. AIC increased to 1445.16.

Step 4: Preoperative hydrocephalus model

Introducing preoperative hydrocephalus as an interaction effect with timepoint showed no significant impact, with the tumour location impact remaining: LCH: *B* = −5.36, SE = 2.13, 95% CI −9.58, −1.13, *p* < 0.05 and FV: *B* = −4.81, SE = 2.38, 95% CI −9.50, −0.11, *p* < 0.05. AIC increased to 1445.16.

Step 5: Sex, age, dysarthria and test format, full model

Adding sex, age, dysarthria and test format reduced the AIC to 1420.36. The interaction of sex with timepoint showed a significant positive effect on postoperative outcomes, indicating that boys had an increase in correct words per minute compared to girls from pre− to postoperative performance (*B* = 4.17, SE = 1.98, 95% CI 0.27 to 8.28, *p* < 0.005). Additionally, there was a significant main effect for test format, with paper format negatively impacting performance compared to screen format (*B* = −5.52, SE = 1.84, 95% CI −9.08, −1.96, *p* < 0.001). Age between 7 and 12 also had a significant positive impact, with children in this age range performing better than those younger or older (*B* = 1.05, SE = 0.63, 95% CI −9.16 to −1.89, *p* < 0.05). Test format and age did not show an interaction with timepoint, indicating that their effects were consistent before and after the operation. This means that these factors had a stable impact on performance regardless of the surgical intervention. The models are presented in table 2.

**Table 2:** Linear mixed model investigating factors affecting word-finding ability

	Step 2	Step 3	Step 4	Step 5
	Model with tumour location	Model with tumour location and histology	Model with tumour location, histology and preop. hydrocephalus	Full model (as step 4 but sex, age, dysarthria and test format added)
	Estimates (95% CI) *p*-value	Estimates (95% CI) *p*-value	Estimates (95% CI) *p*-value	Estimates (95% CI) *p*-value
	(*n* = 176, 266 obs.)	(*n* = 158, 240 obs.)	(*n* = 156, 236 obs.)	(*n* = 137, 200 obs.)
**Postop** (interaction effects)				
Location				
LCH	−5.22 (−9.06 to −1.38) *p <* 0.01	−5.47 (−9.62 to −1.31) *p* < 0.05	−5.36 (9.58 to −1.13) *p <* 0.05	−3.10 (−7.57 to 1.37) *p* 0.179
RCH	1.72 (−1.98 to 5.43) *p* 0.360	0.56 (−3.45 to 4.56) *p* 0.785	0.28 (−3.78 to 4.33) *p* 0.893	0.77 (−3.47 to 5.02) *p* 0.720
BS	−2.17 (−6.91 to 2.57) *p* 0.368	−2.50 (−7.82 to 2.83) *p* 0.357	−1.97 (−7.46 to 3.51) *p* 0.479	−0.86 (−6.53 to 4.81) *p* 0.764
VR	−2.09 (−5.83 to 1.66) *p* 0.273	−2.17 (−6.27 to 1.93) *p* 0.298	−1.78 (8−5.98 to 2.41) *p* 0.403	−1.01 (−5.39 to 3.37) *p* 0.649
FV	−5.17 (−8.90 to −1.44) *p <* 0.01	−4.94 (−9.53 to −0.35) *p* < 0.05	−4.81 (−9.50 to −0.11) *p <* 0.05	−2.57 (−7.47 to 2.33) *p* 0.302
Histology				
MB	−	−0.22 (−5.20 to 4.77) *p* 0.932	−0.06 (−5.13 to 5.01) *p* 0.982	−0.39 (−5.77 to 4.99) *p* 0.887
Other tumour		−0.43 (−5.92 to 5.06) *p* 0.879	−0.11 (8−5.64 to 5.42) *p* 0.968	1.24 (−4.57 to 7.05) *p* 0.674
Preop hydrocephalus; present	−	−	0.34 (−4.04 to 4.72) *p* 0.879	−0.20 (−4.61 to 4.21) *p* 0.928
Sex, boys	−	−	−	4.17 (0.27 to 8.28) *p* < 0.05
**Pre- and postop **(main effects)				
Age				
<7	−	−	−	1.31 (−3.83 to 6.46) *p* 0.615
7-12	−	−	−	1.05 (−0.19 to 2.29) *p* 0.095
>12	−	−	−	−0.13 (−1.62 to 1.36) *p* 0.868
Dysarthria; present	−	−	−	−0.04 (−7.12 to 7.03) *p* 0.991
Test format; paper	−	−	−	−5.52 (−9.16 to −1.89) *p <* 0.005

*LCH* left cerebellar hemisphere, *RCH* right cerebellar hemisphere, *BS* brainstem, *VR* vermis, *FV* fourth ventricle, *PA* pilocytic astrocytoma, *MB* medulloblastoma

A stepwise backward selection approach was then applied. Nonsignificant effects were removed one by one, ending with a final model including only significant effects. The final model showed a reduction in AIC (1391.5) compared to both the Step 4 model (AIC 1445.2) and the full model (AIC 1420.4), indicating a better fit.

Postoperatively, there was a significant negative effect of tumour location in the fourth ventricle (*B* = −4.09, SE = 1.91, 95% CI −7.84, −0.35, *p* < 0.05) and a significant positive effect of male sex, with boys improving more after surgery than girls (*B* = 3.90, SE = 1.75, 95% CI 0.42, 7.38, *p* < 0.05). In addition, there were factors that had similar effects on the outcome before and after surgery. Dysarthria had a significant negative effect (*B* = −4.07, SE = 1.94, 95% CI −7.89, −0.25, *p* < 0.05), as did paper-based test format (*B* = −5.70, SE = 1.34, 95% CI −8.34, −3.06, *p* < 0.001). Additionally, age between 7 and 12 years had a significant positive effect compared to both higher and lower ages (*B* = 1.43, SE = 0.29, 95% CI 0.86, 2.00, *p* < 0.001). The final model is presented in Table 3.

**Table 3:** Linear mixed model investigating factors affecting word-finding ability. Final model including only significant effects

	Estimates	95% CI	*p*-value
**Postop** (interaction effects)			
Location: FV	−4.09	−7.84 to −0.35	**<0.05**
Sex: boys	3.90	0.42 to 7.38	**<0.05**
**Pre- and postop** (main effects)			
Age: 7–12 years	1.43	0.86 to 2.00	**<0.001**
Dysarthria: present	−4.07	−7.89 to −0.25	**<0.005**
Test format: paper	−5.70	−8.34 to −3.06	**<0.001**
Observations 221			

*FV* fourth ventricle

## Discussion

### Postoperative word-finding difficulties and changes in word-finding ability

Comparing postoperative performance with norms from typically developing children in the subset of Swedish children, we found that these children were approximately twice as slow after surgery compared with the established norms, with the majority of test times being two standard deviations above the Swedish norms (i.e. substantially slower). A previous study had indicated a relatively low incidence of word-finding difficulties after PFT surgery (12%), but the word-finding test used in this previous study did not measure speed and is thus likely to have underestimated the frequency of postoperative word-finding difficulties. Our current findings suggest that speed may represent a major concern, contributing significantly to postoperative word-finding difficulties.

Compared to norms from typical development, we also found severe word-finding difficulties before surgery, suggesting a negative impact of the tumour itself (cf. Persson et al. 2024). This opens the possibility that the postoperative word-finding difficulties we identified were present already before surgery, and indeed, when we directly compared performance before and after surgery in the full crosslinguistic sample, we found no significant group-level difference between pre- and postoperative word-finding abilities. While this lack of group-level difference could have reflected that children’s poor word-finding abilities simply persisted after surgery, with no effect of surgery, close inspection of individual data, both in the Swedish subsample and in the full sample, reveals that the lack of group-level difference in fact reflects substantial variability in outcomes among individual children. Some children exhibited noticeably improved word-finding abilities and others showed marked decline after surgery. The finding that PFT surgery leads to improved word-finding abilities for some children aligns with the results from di Rocco et al. [22]. Our study further replicates previous findings that even children without POSI after surgery for PFT may exhibit postoperative word-finding difficulties [10]. We found no significant association between the presence of POSI and postoperative decline in word-finding ability. For mutism, on the other hand, the results suggest an association, as all four children with a period of mutism showed a decline in word-finding ability. Such an association would support the idea of these difficulties varying along a continuum in severity, but note that this observation is based on only four children.

### Factors affecting postoperative word-finding ability

The wide variability in postoperative outcomes, with some children exhibiting improved word-finding abilities after surgery and others experiencing deterioration, makes it important to examine the factors affecting these different outcomes. We did not find support for our hypothesis that the involvement of the right cerebellar hemisphere and brainstem increased the risk of postoperative word-finding difficulties, as seen preoperatively [21]. We did, however, find support for the hypothesis of a negative effect of tumour location in the fourth ventricle on postoperative word-finding ability compared with preoperative performance, indicating that the surgical removal of fourth-ventricle tumours leads to poorer word-finding ability. This aligns with the finding by Svaldi et al., who in their review of individual patient studies found that nearly 86% of children with fourth-ventricle tumours (12 out of 14) had postoperative language impairment [24]. The roof and walls of the fourth ventricle contain key elements of the dentato-thalamo-cortical pathway (DTCP), which is the main efferent pathway crucial to cerebellar involvement in higher cognitive functions, including language [26, 46]. Damage to this pathway, encompassing the superior cerebellar peduncle inserting on the brainstem, has also been associated with a higher risk of POSI [3, 29]. Di Rocco et al. also found that tumours involving the dentato-thalamo-cortical pathway (DCTP) contribute to postoperative language impairments, particularly through their role in disrupting cerebellar functions critical for language processing [8]. It is thus highly likely that the disruption of the DTCP contributes to postoperative word-finding difficulties. Surgical approach was not in focus in the current study, as our previous prospective multicenter cohort study of posterior fossa tumours in children (cf. Grønbæk et al. 2021), which represents the largest study to date, did not identify a significant association between surgical approach and POSI [3]. However, these associations remain debated with contradictory findings [3, 31–36]. We found an effect of sex, with boys performing better after surgery. In a previous study of preoperative word-finding difficulties, we found a higher prevalence of boys with PFT experiencing word-finding difficulties [21]; therefore, one possible explanation for this sex-specific effect is that tumour removal has the potential to enhance word-finding abilities more in boys because the tumour in itself was causing more word-finding difficulties in boys. There were no differences in performance between sexes in the normative material [42]. Neither histology nor preoperative hydrocephalus were found to affect postoperative outcomes. Molecular profiling data, which distinguishes medulloblastomas into subgroups with differing biological profiles and risks, was not consistently available in our cohort due to variations in its implementation across centres. Consequently, molecular subgrouping was not included in this study. Given that wingless, group 3 and group 4 are associated with a higher risk of CMS, this data could have offered insights into whether subgroup-specific tumour biology plays a role in the severity or type of postoperative word-finding difficulties [30]. Children between 7 and 12 years of age were found to perform significantly better compared to both younger and older children, but this age effect was the same before and after surgery, i.e. it was not related to surgery. As expected, the presence of dysarthria and paper-based test format were also associated with poorer word-finding performance, but again, these effects were constant across pre- and postoperative performance.

### Limitations and future directions

An important limitation is that our instrument for evaluating word-finding abilities, Wordrace, lacks norms for other languages than Swedish. Consequently, comparison with typical developmental trajectories is feasible only for the Swedish children. The present study only investigates word-finding abilities 1–4 weeks after surgery. To understand the long-term effects of surgery for PFT on language development and to identify factors that contribute to the persistence or improvement of word-finding difficulties over time, longitudinal studies would be highly valuable. The variability in the timing of postoperative assessments, conducted between 1 and 4 weeks after surgery, is another limitation. According to the original study design, speech recordings were ideally intended to be conducted within two weeks of surgery; however, in cases of logistical challenges or difficult patient scenarios, recordings were permitted up to four weeks after surgery. Differences in recovery phases, including the potential onset or improvement of cerebellar mutism during this period, could influence word-finding abilities and affect the consistency of the results

The included participants are not fully representative, as children who remained mute beyond the 1–4-week postoperative assessment period were not included in the analysis. When comparing the included children with those with missing speech data, we observe notable differences in the incidence of postoperative dysarthria and postoperative hydrocephalus. A higher proportion of children in the missing data group show postoperative dysarthria compared to the included children. The exclusions may have contributed to underestimation of postoperative word-finding difficulties in the study population. The lack of data on tumour volumes limits the evaluation of the influence of size on postoperative word-finding difficulties. Future studies with tumour volume measurements could clarify its potential impact. Furthermore, dysarthria was not assessed by speech-language pathologists, and therefore, the results should be interpreted with caution. Future studies are encouraged to focus on motor speech impairments with auditory-perceptual speech analysis. Additionally, the study protocol provided information about the areas encroached on by the tumour, but it did not specify the main location or the extent of tumour infiltration in each listed location. This lack of precise information limits the conclusions that can be drawn regarding associations between tumour location and word-finding difficulties.

## Conclusions

This study contributes to our understanding of postoperative word-finding difficulties in children undergoing PFT surgery. The principal finding is that word-finding difficulties are prevalent in the postoperative phase, also without being preceded by POSI, with word-finding speed as the primary concern. Tumour location in the fourth ventricle had a significant negative effect on postoperative word-finding abilities, indicating that the surgical removal of fourth-ventricle tumours leads to poorer word-finding ability. The frequency of word-finding difficulties not preceded by POSI underscores the need for targeted assessments and interventions in the postoperative phase for children undergoing PFT surgery. Enhancing children’s word-finding abilities is crucial, as difficulties in this area can affect their academic skills, social participation and overall well-being.

## Data Availability

Due to ethical considerations, data associated with this paper cannot be accessed.
